# Comparative analysis of bonding strength between enamel and overlay of varying thicknesses following an aging test

**DOI:** 10.1016/j.jds.2023.06.011

**Published:** 2023-06-22

**Authors:** Chih-Wei Tseng, Chen-Yin Yong, Chih-Yuan Fang, Sheng-Yang Lee, Yu-Chieh Wang, Wei-Chun Lin

**Affiliations:** a Department of Dentistry, Wan Fang Hospital, Taipei Medical University, Taipei, Taiwan; b School of Dentistry, College of Oral Medicine, Taipei Medical University, Taipei, Taiwan; c Center for Tooth Bank and Dental Stem Cell Technology, Taipei Medical University, Taipei, Taiwan; d School of Dental Technology, College of Oral Medicine, Taipei Medical University, Taipei, Taiwan

**Keywords:** Overlay, Bonded strength, Aging test, Enamel, Natural teeth, Dental restoration

## Abstract

**Abstract:**

*Background/purpose:* Overlay restorations can be used clinically as a treatment option to preserve natural dentine. However, whether the residual enamel thickness and overlay thickness affect the adhesion between the restoration and tooth is still unknown. This study was to investigate effects of the overlay thickness and residual enamel thickness on bonding strength.

**Materials and methods:**

Overlays of different thicknesses were prepared with natural teeth which had 2, 4, and 6 mm of occlusal reduction (n = 10). Specimens were subjected to 10,000 cycles in water at 5–55 °C, and finally compressive strength tests were used to evaluate the bonding strength.

**Results:**

All groups showed good bond strength (*P* > 0.05). The overlay restorations of different thicknesses reduced the preparation amount by 30.3%–7.2% and significantly preserved more of the tooth structure (*P* < 0.005). Compared to the control group, the overlay restoration increased the marginal fitness by about 0.67–0.88 times. The thermal cycling indicated that the decrease in the maximum bearing stress was due to the aging of the ceramic itself. Therefore, the thickness of the overlay had a greater influence on the compressive strength than the bond strength.

**Conclusion:**

Based on the above this study recommends an overlay thickness of at least 2 mm in clinical practice. The aging test confirmed that adhesion between the overlay and teeth was quite firm and stable. This shows that a stable adhesive effect of the overlay can be used as a treatment option for preserving a greater amount of a tooth's structure.

## Introduction

Dental caries is one of the most common dental problems in dentistry. A retention form or overall preparation is necessary for traditional prosthodontic treatment, such as an inlay, onlay, or full crown. Previous studies showed that the loss of a tooth's structure, by caries or by preparation, can weaken the structure of the tooth, which implies more-conservative procedures may be required for a tooth's preparation.^1^, ^2^, ^3^, ^4^ A conservative prosthetic design, known as an overlay, for restoring occlusal and esthetic functions has become practical and more popular in recent years.^3^^,^^4^ An overlay is a partial-coverage restoration which covers the entire occlusal surface of a tooth.^5^ Due to the strength of the adhesive and bonding procedures, an overlay can retain its position and function on the tooth without a retention form. However, the success of adhesive prostheses is dependent on the residual enamel.^6^, ^7^, ^8^ The bonding strength of an overlay is directly related to the interaction conditions of the bonding surface between the tooth surface and restorative material.^2^^,^^3^^,^^9^ Although an in vitro study was conducted to understand the bonding nature of adhesive and bonding systems,^10^ the minimal amount of enamel to successfully support an adhesive prosthesis or overlay is still unclear so far. There are also no recommendations or guidelines for pre-evaluation and tooth preparation before applying a ceramic overlay. This may be due to the complexity of clinical scenarios. Too many clinical variables can complicate matters, with several common examples being the total area of the enamel, chewing pattern, the direction of the chewing force, and the height of the prosthesis.^11^^,^^12^

In addition, aging tests of the adhesive strength of dental restorations have also attracted much attention. Because of the complexity of the oral environment, changes such as cold, heat, and acid occur during the process of ingesting food. These factors can affect the bonding surfaces between a dental restoration and a tooth, reducing their long-term stability.^13^ In order to make the test results of materials more consistent with clinical results, many studies simulate the environment in the oral cavity through thermal cycles between hot and cold.^14^^,^^15^ This helped assess the long-term utility of the adhesive strength between the overlay and residual enamel through the aging test.^16^

In order to gain a further understanding of this issue, this study used compression force to test the limitation of the overlay with different occlusal reduction volumes. The durability of the overlay adhesion was evaluated by simulated aging tests with hot and cold cycles. From this study, the benefit from the residual enamel and the failure mode of the overlay with different heights could be determined. The tested null hypotheses of this study were: (1) Different thicknesses of the overlay on the enamel will not be dislodged. (2) The overlay can still be used as a treatment option for clinical caries restoration after the aging cycle test.

## Materials and methods

**Figure 1:**
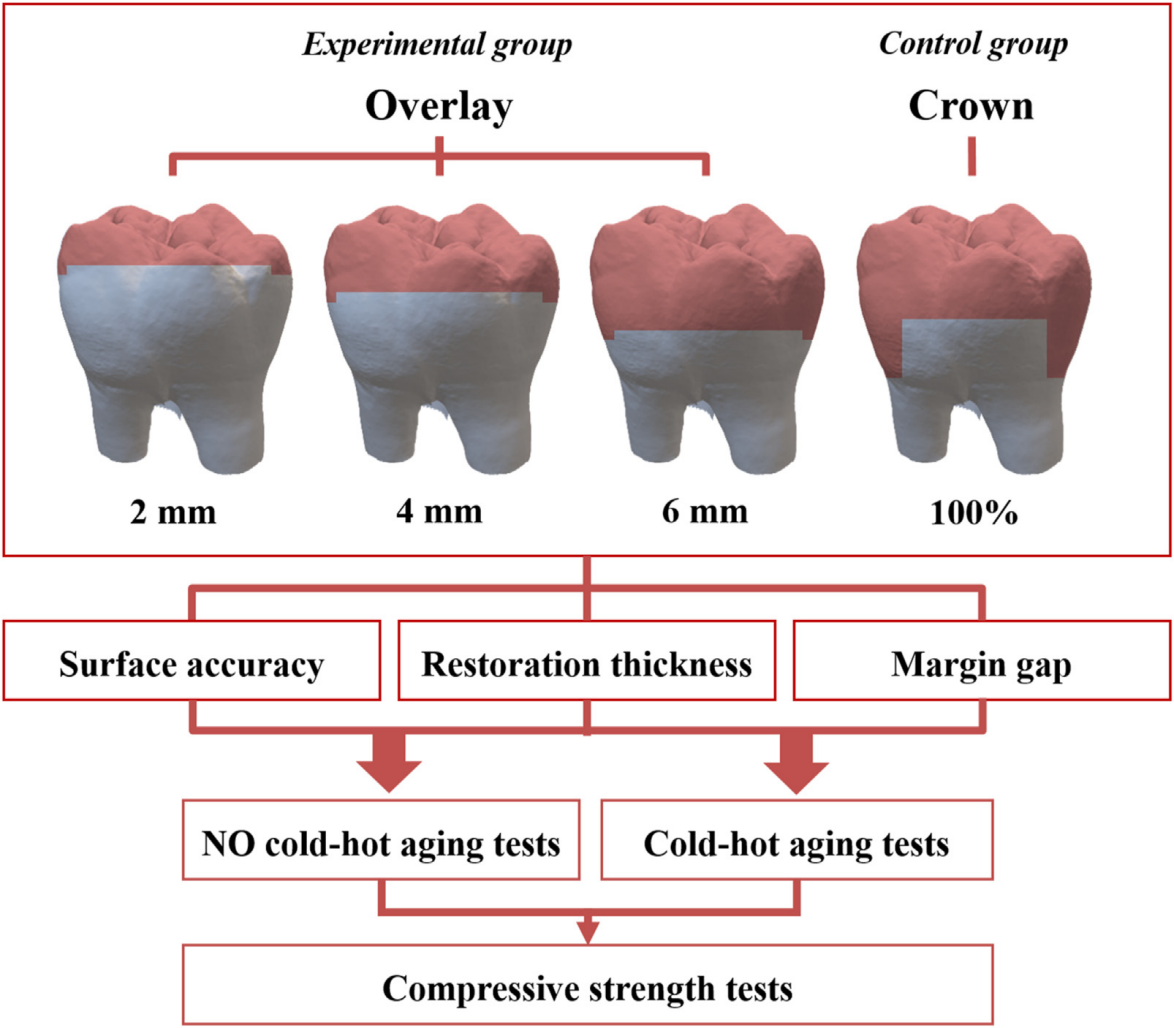
Experimental design and procedures.

The study was conducted in accordance with ethical principles of the *Declaration of Helsinki*. Ethical approval was granted by the Taipei Medical University Joint Institutional Review Board, Taiwan (TMU-JIRB no: N202112033). In this study, 40 human maxillary and mandibular molar teeth extracted due to treatment of orthodontics or periodontal disease were collected as research samples. First, the teeth were scanned on a desktop dental scanner (Medit T510, Medit, Seoul, South Korea). The teeth were prepared with a specific occlusal reduction and scanned again. Two digital models were superimposed to confirm the thickness of the occlusal reduction. The teeth were randomly divided into three groups with different thicknesses of occlusal reduction (2, 4, and 6 mm), with each group containing 10 test samples each. Teeth with a crown preparation were used as the control group (Fig. 1).

After tooth preparation, the overlay and crown restorations were designed with inLab computer-assisted design (CAD)/computer-assisted manufacturing (CAM) dental design software (inLab WS16, Dentsply Sirona, North Carolina State, NC, USA). All restorations were created from lithium disilicate (Mark II, VITA, Säckinge, Germany) in a CAD/CAM dental engraving machine (MCXL, Dentsply Sirona). Subsequently, the inner surface of the ceramic restoration was treated with 5% hydrofluoric acid for 20 s, and cleaned with ultrasonic vibrations in water twice for 5 min each, followed by air-drying. The restoration was then silanized (Monobond Plus, Ivoclar, Zurich, Switzerland) for 60 s, followed by air-drying. Natural teeth were etched with 37% phosphoric acid (enamel: 30 s, dentin: 10 s, Gel Etchant). A primer (OptiBond FL Prime, KaVo Kerr, Brea, CA, USA) was then applied for 15 s with blow-drying. Resin cement (OptiBond FL Prime, KaVo Kerr) was applied to the ceramic surface, which was then seated onto the tooth.^13^

To evaluate the enamel distribution of natural teeth with 2, 4, 6 mm of occlusal reduction and a full-crown preparation, teeth were cut with a low-speed cutter (CL40, Top Tech, Taichung, Taiwan), and the enamel thickness was subsequently measured with an optical microscope (S9D, Leica, Wetzlar, Germany). All teeth were scanned using a desktop scanner. After cementation, the teeth were scanned again and then compared to the original data using analytical software (Medit Compare, Medit) to analyze the surface trueness.^17^ To accurately measure the thickness of the restoration, digital images of before and after cementation were superimposed and analyzed by analytical software (Medit Compare, Medit). The restoration thickness was measured at three different sites on both the transverse and longitudinal sections using automatic vertical measuring software. The margin gap of the restoration was measured through an optical microscope. Marginal gaps were measured at four sites (buccal, lingual, mesial, and distal) of the restorations at a magnification of 2.5 times, and gaps were measured at three points on each side; the mean marginal fit was ultimately calculated.

Samples were immersed in a water tank at alternating temperatures of 5 and 55 °C using a double tank thermal impact machine (TBN-971105, TEN Billion, Tainan, Taiwan).^18^ Each temperature was maintained for 30 s and the transfer time between temperature cycles was 5 s; this was repeated for 10,000 cycles each time (a total of 180.5 h). After the aging test was completed, the shear test was again performed. All of the teeth were embedded in self-curing acrylic resin (Implacryl, Vertex Dental, Zeist, Netherlands) below the cementoenamel junction.^19^ Samples were then placed on a universal testing machine (AGX-V, Shidadzu, Tokyo, Japan), and a compression tester provided by the manufacturer was used to press down from the crown to the apex at a speed of 1 mm/min until the restoration fractured. The maximum pressure was recorded and statistical analyses were performed. In addition, the fracture of the sample was evaluated through optical microscopic and digital software scanning.

All data are expressed as the mean ± standard deviation from 10 replicates. This study used JMP 16 software (Statistics Analysis System, North Carolina State, NC, USA) for data analysis. Significant differences in data were assessed by a one-way analysis of variance (ANOVA) with Tukey's post-hoc test, where *P* < 0.05 was considered significant.

## Results

**Figure 2:**
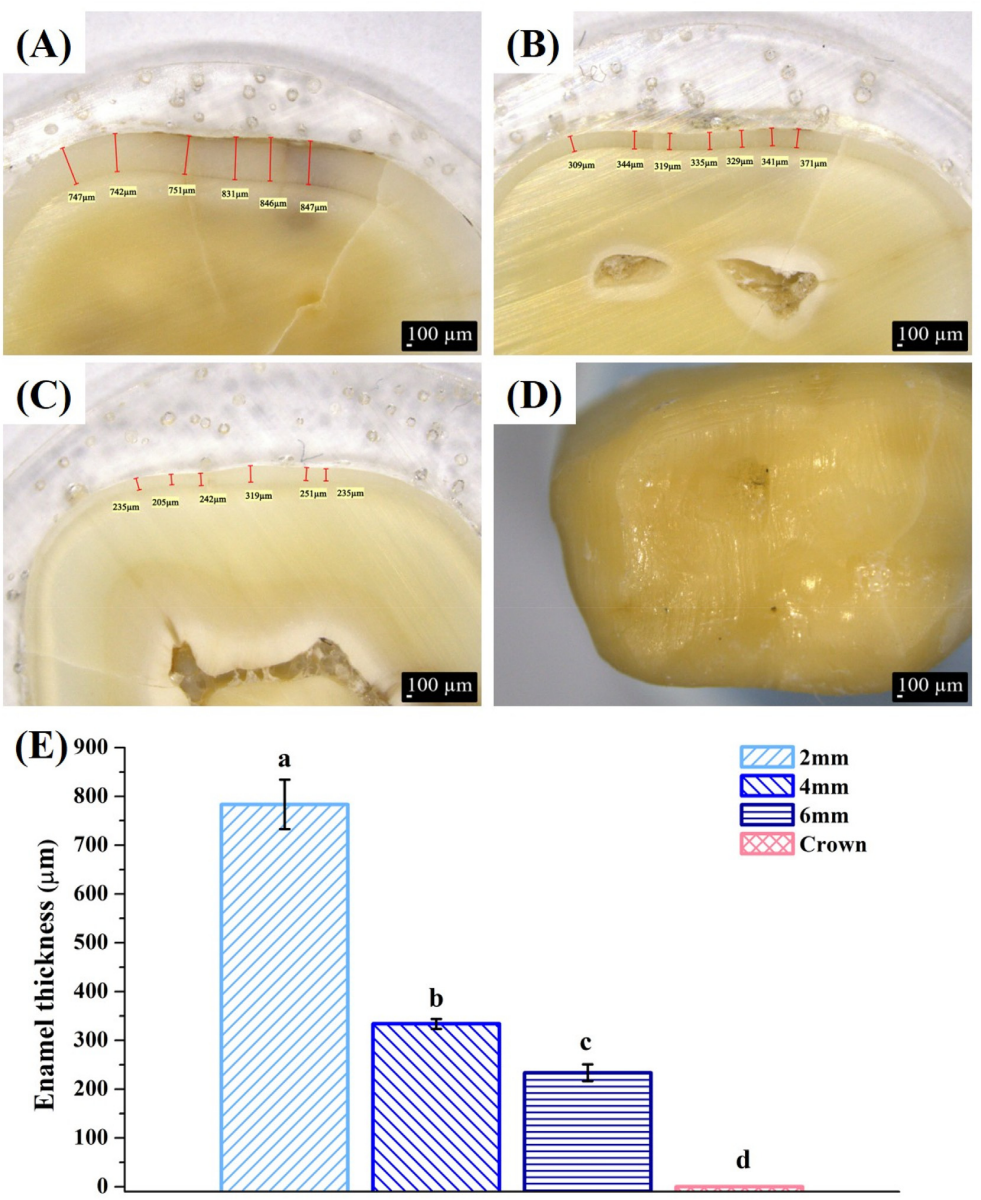
Enamel thickness of natural teeth at different positions. The 2 mm (A), 4 mm (B), 6 mm (C) and full crown (D). Calculation of the thickness of the enamel at different positions of the tooth (E). Means with different letters were significantly different (*P* < 0.05, mean ± SD, n = 5).

In this study, the enamel thickness with 2-mm group (783.4 μm) was the thickest, followed by 4 mm group (333.6 μm), 6 mm group (233.6 μm), and crown preparation (0 μm, Fig. 2). Calculation of the mean RMSE of each group showed that values with 2-mm and 4-mm group were significantly smaller than those of the other groups (*P* < 0.05) (Table 1 and Fig. 4A). Thicknesses of the restorations were 2.2 mm (2-mm), 4.4 mm (4-mm) and 5.9 mm (6-mm), and there were statistical differences among the groups (*P* < 0.05). The marginal gaps after cementation in all groups were measured with an optical microscope (Fig. 5). The 2-mm group had the smallest gap (37.2–46.2 μm), and there was a significant difference (*P* < 0.05). Gaps in the other groups were 38.4–51.2 μm (4-mm group), 48.4–54.4 μm (6-mm group), and 56.4–65.4 μm (control group) (Fig. 6). Damage to the restorations by the compressive test was evaluated by comparing differences in surface accuracy using analytical software. Results showed that mean RMSE values of all groups before the aging test were 81 μm–432 μm. Mean RMSE values of samples after the aging test were 98 μm–512 μm. This shows the aging test increased the RMSE values (Table 1).

**Table 1:** The shape difference of the restoration at different testing stages.

Mean RMSE (μm)	Original	Compressive strength tests	
		Before aging tests	After aging tests
2 mm	287 (±18)^b,A^	122 (±13)^b,C^	158 (±18)^b,B^
4 mm	298 (±17)^b,A^	108 (±15)^b,B^	98 (±12)^c,B^
6 mm	349 (±31)^a,A^	81 (±12)^c,C^	128 (±10)^c,B^
Crown	374 (±29)^a,C^	432 (±31)^a,B^	512 (±33)^a,A^

Mean RMSE: the mean root mean squared error (RMSE) represents the overall surface difference of the sample.

Original: the original control group was natural teeth without grinding.

Compressive strength tests: the control group for compressive strength tests was the tooth and restoration before the test.

Means with different letters were significantly different (*P* < 0.05, mean ± SD, n = 10).

According to the stress-strain curve, the curve became higher as the thickness of the overlay increased (Fig. 8A). Compression curves of all groups decreased after the aging test (Fig. 8B). The maximum bearing forces of all groups are shown in Fig. 8C. Before the aging test, the bearing force of the 2-mm group was 1241.32 N, which was significantly smaller than those of the other groups (*P* < 0.05). Bearing forces of other groups were 1425.38 N (4-mm group), 1494.41 N (6-mm group), and 1545.74 N (control group), and there were no differences among them (*P* > 0.05). After the aging test, the maximum bearing forces were 834.54 N (2-mm group), 836.09 N (4-mm group), 917.55 N (6-mm group), and 1216.94 N (control group). The bearing force of the control group was significantly higher than those of the other groups (*P* < 0.05). The bearing strengths of all specimens were ultimately significantly reduced after the aging test (*P* < 0.05).

## Discussion

In this study, a ceramic overlay was proposed as an alternative treatment to crown restorations, avoiding extensive tooth structure preparation to preserve as much of the healthy tooth structure as possible during caries treatment. In this study, natural teeth were prepared with different occlusal reductions, and the enamel thickness was subsequently measured using a light microscope. Results showed the thickness of the enamel gradually decreased as it extended toward the root. The crown restorations in the control group left only the dentin structure (Fig. 2).

**Figure 3:**
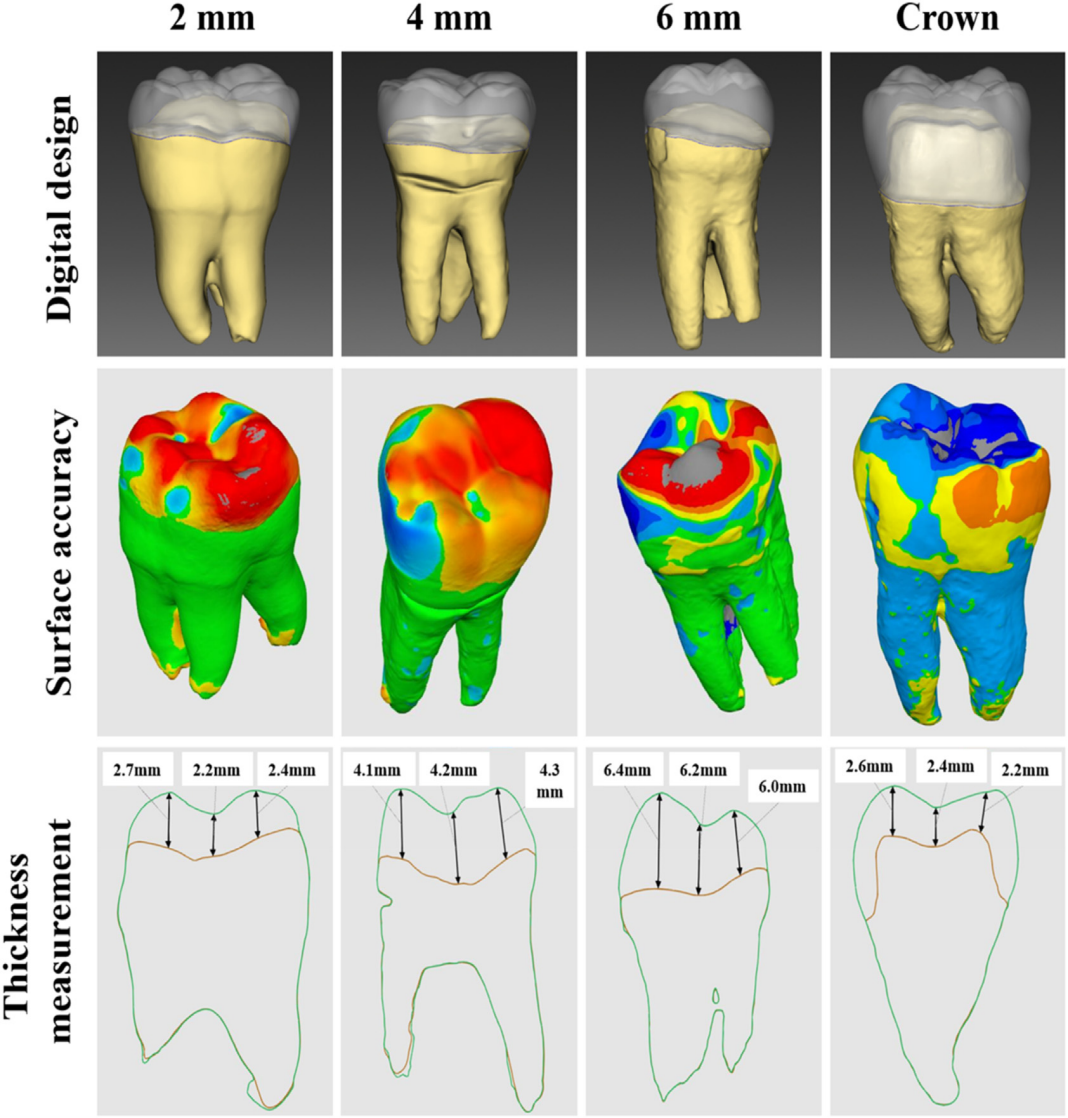
Digital design of natural tooth preparation and measurement of restoration thickness.

**Figure 4:**
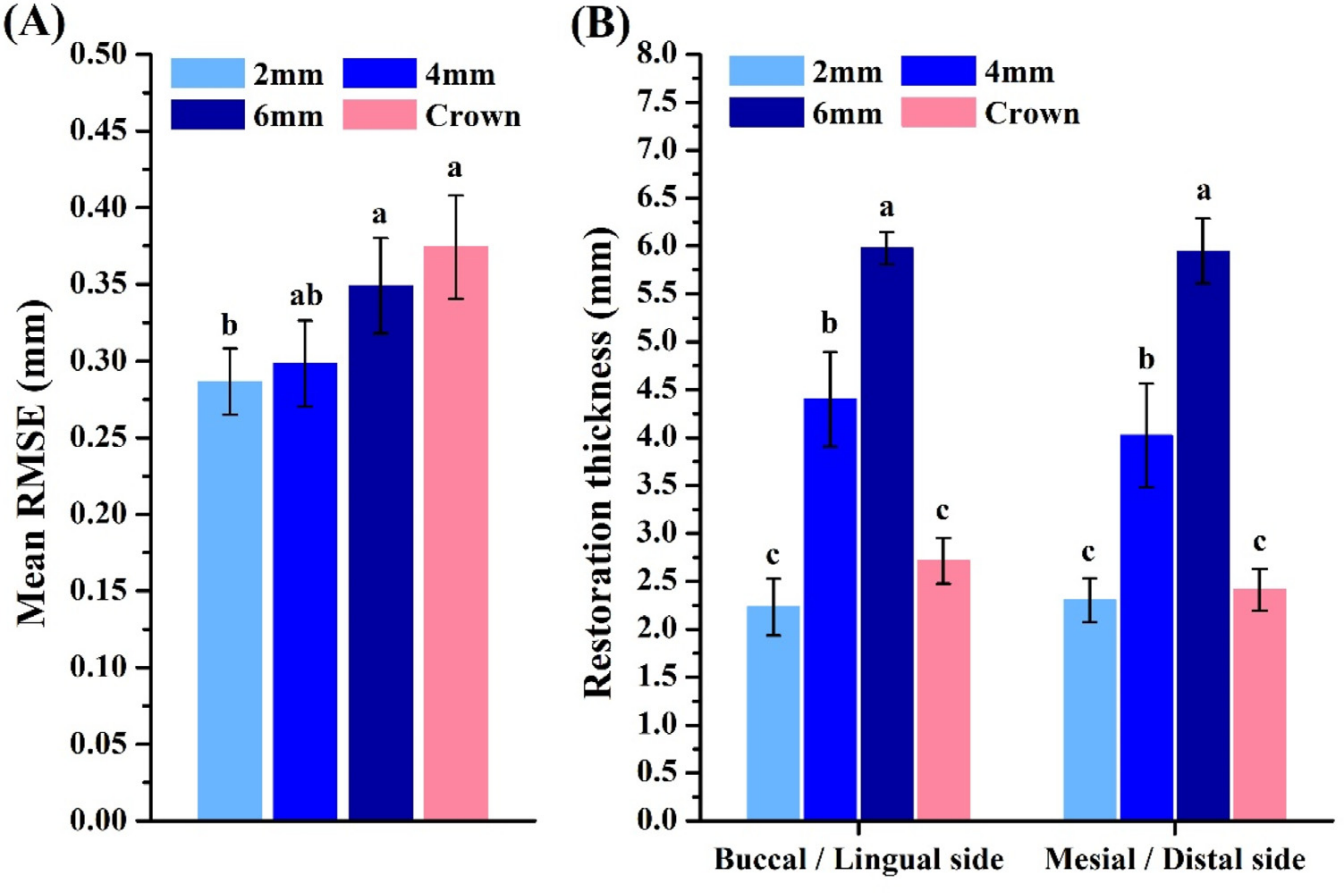
Restoration shape differences and thickness measurement. The shape difference (A) and measurement of thickness (B). Means with different letters were significantly different (*P* < 0.05, mean ± SD, n = 10).

The surface trueness results showed that the overlay was less damaging to the tooth structure than the crown preparation (Fig. 3). A quantitative analysis of the surface accuracy by calculating the RMSE (mm) showed that it was largest in the crown (Table 1). This means that the RMSE was positively correlated with the amount of tooth preparation. Compared to the control group, overlays with different occlusal reductions reduced preparation amounts by 30.3% (2-mm group), 25.5% (4-mm group), and 7.2% (6-mm group) (Fig. 4A). Therefore, it was shown that an overlay restoration can retain more of the true appearance of the natural teeth and avoid excessive sacrifice of a tooth's structure. The scanners were equipped with digital software that could analyze the cross-section of the scanned object (Fig. 3). The thickness of the restorations was measured with analytical software. Results showed that the restoration thicknesses of all overlay groups were consistent with the experimental design (Fig. 4B). The average thickness of the control ranged 2–2.5 mm. In clinical practice, a full-crown preparation requires at least 1.5 mm of occlusal reduction, which can then provide sufficient strength.^20^ In this study, all restoration specimens had a thickness greater than the minimum thickness required for ceramic materials, reducing the effects of material factors in the study.

**Figure 5:**
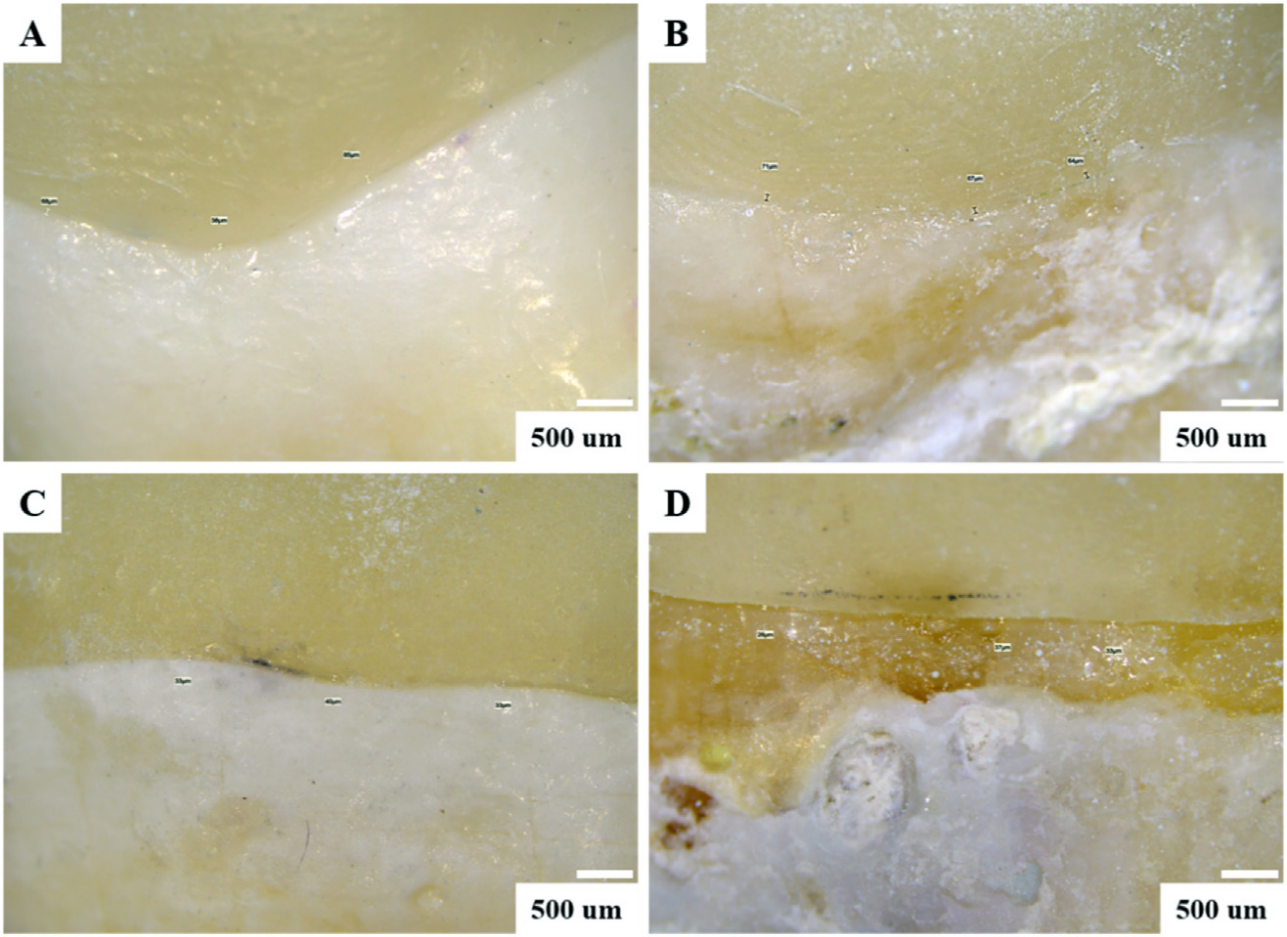
Optical images of restorations bonded to natural teeth.

**Figure 6:**
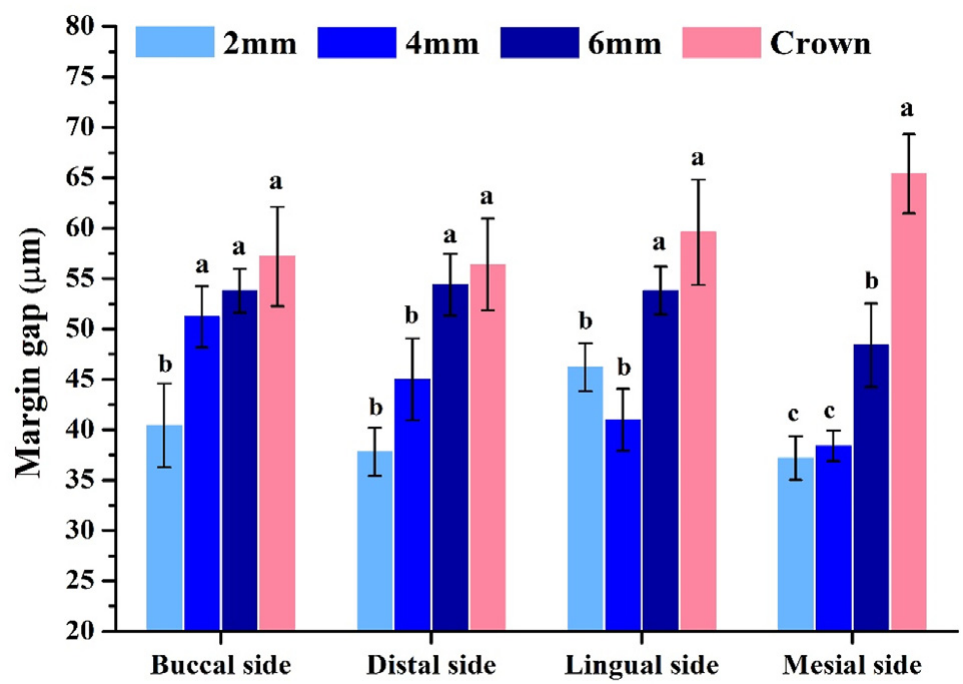
Margin gap measurement after the restoration is bonded to the natural tooth. Means with different letters were significantly different (*P* < 0.05, mean ± SD, n = 10).

The marginal fitness between the restoration and tooth is important to avoid secondary caries.^21^^,^^22^ This study observed the marginal gap between the overlay and crown after cementation through optical microscopy. Results showed that the restorations of all groups had an excellent marginal fit (Fig. 5). The overlay groups had better marginal fits than did the control group, and the 2-mm group was the fittest among these groups. This may have been because of the angle of tooth preparation, which was parallel to the occlusal surface and reduced the complexity. The marginal gaps of all restorations in this study were between 37.2 and 65.4 μm (Fig. 6), which were significantly lower than the minimum clinical requirement of 120 μm.^23^ This indicates that the overlays had better marginal fits than did the crown restoration.

**Figure 7:**
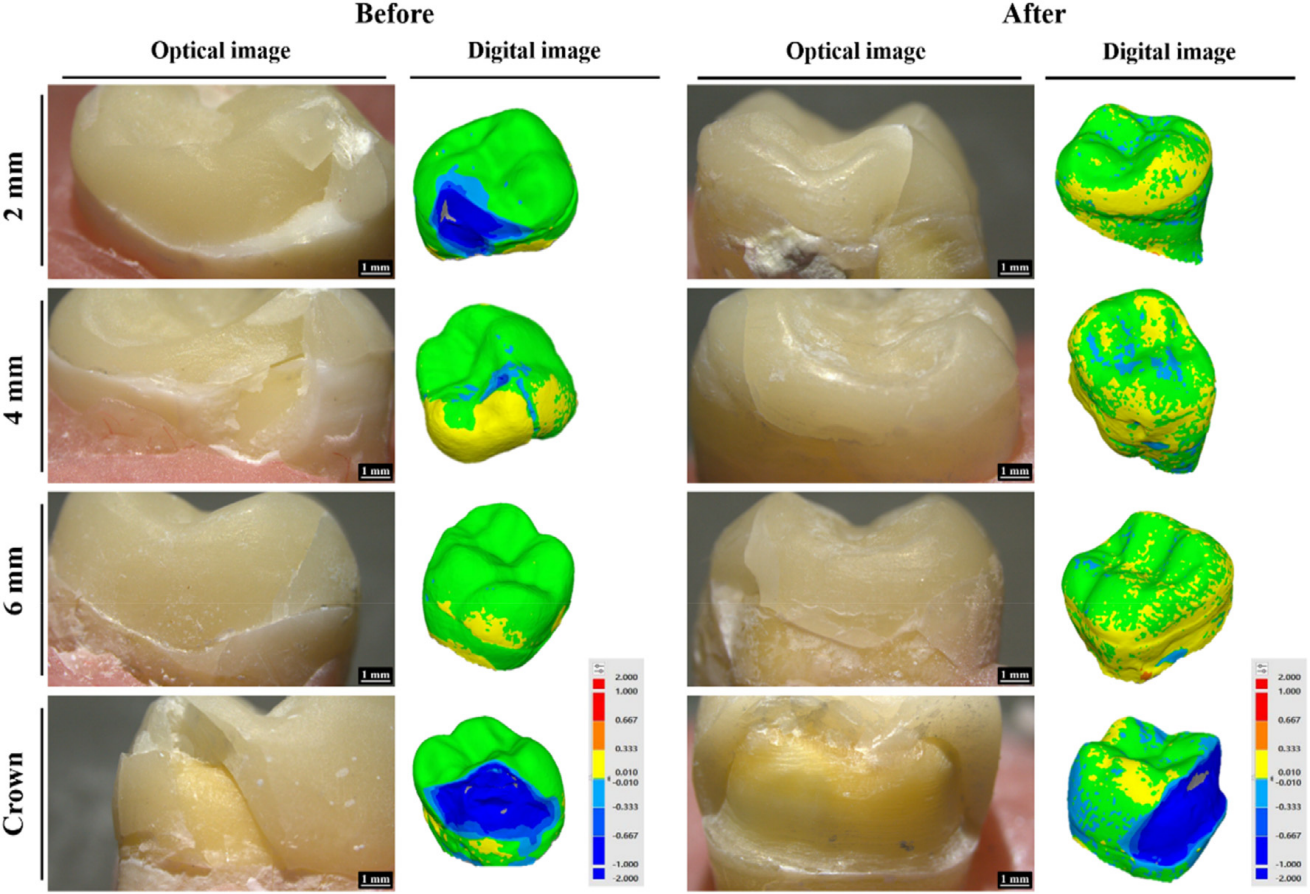
Optical and digital images of the compressive strength of the restoration before and after the aging test.

**Figure 8:**
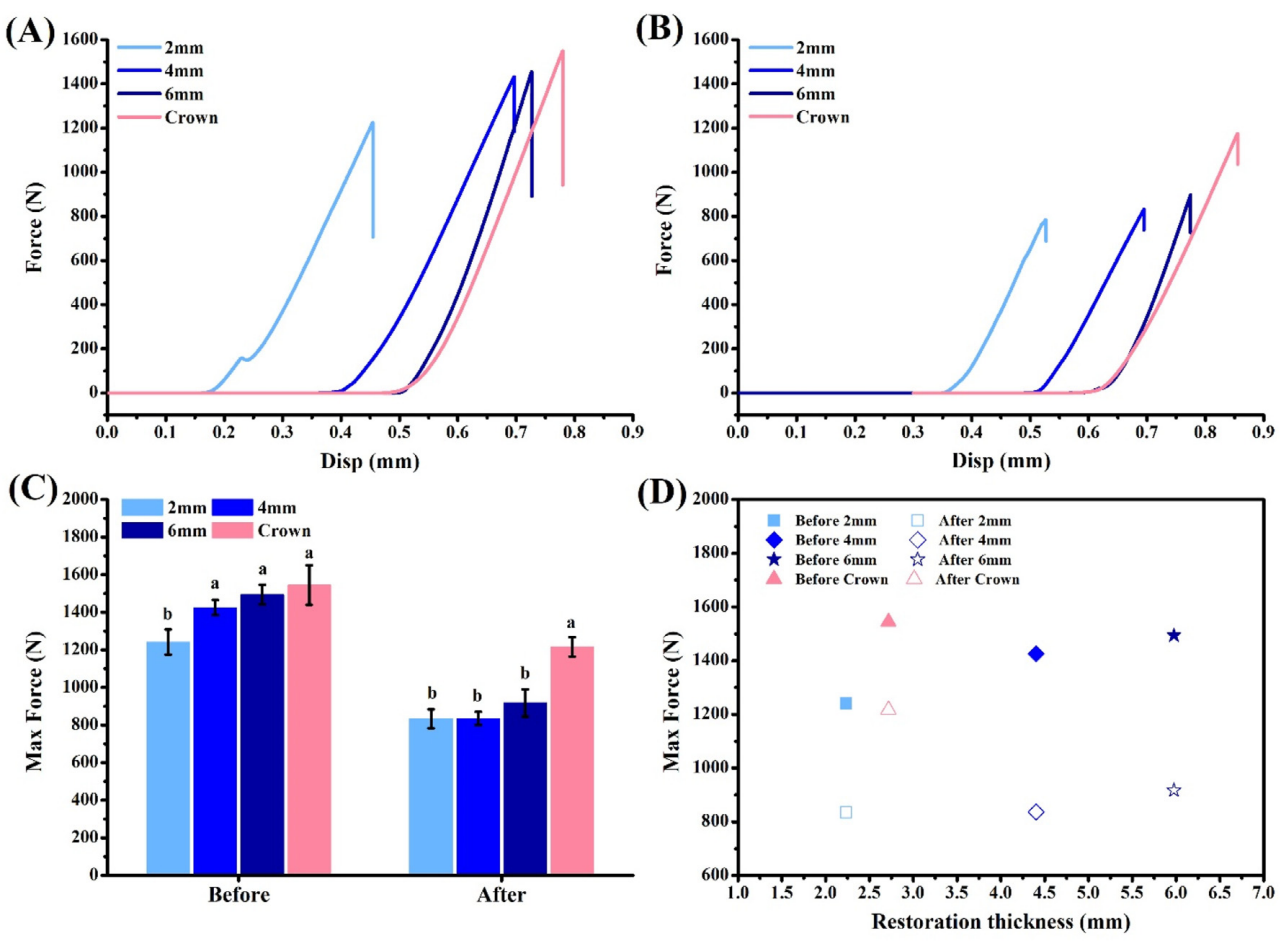
Compressive strength testing of different enamel areas bonded to restorations. Compression curves before (A) and after the thermal aging test (B). The maximum bearing capacity of the restoration (C). Comparison of restoration thickness and maximum bearing force (D). Means with different letters were significantly different (*P* < 0.05, mean ± SD, n = 5).

There are many types of dental restorations, including inlays, onlays, overlays, and crowns. These restorations differ in design, which affects their retention effects and bond strengths.^24^^,^^25^ Nowadays, with advances in adhesive dentistry, the bond strength has improved and has become reliable.^26^^,^^27^ Therefore, bond strengths of all groups were measured through a compressive strength test in this study. Results showed that none of the samples had adhesive failure before or after the aging test (Fig. 7). However, only cracks appeared in the overlay groups in this study, which indicated that the adhesive between the overlay and tooth was not damaged (Fig. 7). The surface accuracy was evaluated after the compressive strength test using analytical software (Table 1). This shows that ceramic restorations in the control group were greatly damaged, especially after the aging test. Analyzing the maximum force borne by the specimens showed that the control group could withstand higher forces (Fig. 8A–C). Comparing the effect of different restoration thicknesses on the maximum bearing pressure (Fig. 8D) showed that the thicker the overlay restoration, the greater the bearing force it could withstand. Although the restoration thickness of the control group was similar to that of the 2-mm overlay group, structural differences increased the bearing force by about 19.7%. In addition, although the crown restoration had a higher bearing force, the hollow inside structure caused a large portion of ceramic fracture to occur. Another factor was the surface of the tooth to which the crown adhered, which is dentin. Previous studies showed that stronger bonding could be achieved on enamel than on dentin.^28^^,^^29^ Therefore, when the ceramic of the crown restoration is damaged, it can cause the restoration to be dislodged.

The destruction caused by compressive strength mainly occurred in the ceramic structure. Therefore, the maximum bearing force dramatically declined after the aging test. The control group exhibited the least decline at about 21.3%, followed by the 2-mm group (32.8%), 6-mm group (38.6%), and 4-mm group (41.3%). This is because the mechanical properties of the ceramic decreased after thermal cycling.^30^^,^^31^ Nevertheless, after simulating a 1-year placement of the restoration in the mouth, the maximum bearing forces of the restorations were still higher than the human occlusal force of 427–632 N.^32^ This means that an overlay restoration would have excellent clinical performance. The results support the two hypotheses of this study: (1) different thicknesses of overlay on the enamel will not cause it to become dislodged; and (2) the overlay can still be used as a treatment option for clinical caries restoration after the aging cycle test.

Within the limits of our study, using an overlay restoration as a treatment option preserves a greater amount of tooth structure than a crown restoration. The aging test confirmed that adhesion between the overlay and teeth was quite firm and stable. Considering the strength of the restoration, it is recommended to have at least 2 mm of overlay thickness. Overlay restorations can be an alternative option for caries treatment in clinical practice.

## Conflicts of interest

The authors have no conflicts of interest relevant to this article.

## References

[1] Taha N.A., Messer H.H. (2016). Restoration of the root-filled tooth. Prim Dent J.

[2] Bedran-Russo A., Leme-Kraus A.A., Vidal C.M.P., Teixeira E.C. (2017). An overview of dental adhesive systems and the dynamic tooth–adhesive interface. Dent Clin North Am.

[3] Naik V.B., Jain A.K., Rao R.D., Naik B.D. (2022). Comparative evaluation of clinical performance of ceramic and resin inlays, onlays, and overlays: a systematic review and meta analysis. J Conserv Dent.

[4] Channarong W., Lohawiboonkij N., Jaleyasuthumkul P., Ketpan K., Duangrattanaprathip N., Wayakanon K. (2022). Fracture resistance of bonded ceramic overlay restorations prepared in various designs. Sci Rep.

[5] Luciano M., Francesca Z., Michela S., Tommaso M., Massimo A. (2020). Lithium disilicate posterior overlays: clinical and biomechanical features. Clin Oral Invest.

[6] Liu J.F., Yang C.C., Luo J.L., Liu Y.C., Yan M., Ding S.J. (2022). Bond strength of self-adhesive resin cements to a high transparency zirconia crown and dentin. J Dent Sci.

[7] Chen K.K., Chen J.H., Wu J.H., Du J.K. (2021). Influence of commercial adhesive with/without silane on the bond strength of resin-based composite repaired within twenty-four hours. J Dent Sci.

[8] Bumrungruan C., Sakoolnamarka R. (2016). Microshear bond strength to dentin of self-adhesive flowable composite compared with total-etch and all-in-one adhesives. J Dent Sci.

[9] Silva e Souza Junior M.H., Carneiro K.G.K., Lobato M.F., Silva e Souza PdAR., Góes MFd (2010). Adhesive systems: important aspects related to their composition and clinical use. J Appl Oral Sci.

[10] Sebold M., André C.B., Sahadi B.O., Breschi L., Giannini M. (2021). Chronological history and current advancements of dental adhesive systems development: a narrative review. J Adhes Sci Technol.

[11] Monteiro R.V., Dos Santos D.M., Bernardon J.K., De Souza G.M. (2020). Effect of surface treatment on the retention of zirconia crowns to tooth structure after aging. J Esthetic Restor Dent.

[12] Dejak B., Młotkowski A. (2020). A comparison of mvm stress of inlays, onlays and endocrowns made from various materials and their bonding with molars in a computer simulation of mastication–FEA. Dent Mater.

[13] Lin S.C., Lin W.C., Hu T.C., Yan M., Tang C.M. (2021). Evaluation of the bonding strength between various dental zirconia models and human teeth for dental posts through in vitro aging tests. Coatings.

[14] Gale M., Darvell B. (1999). Thermal cycling procedures for laboratory testing of dental restorations. J Dent.

[15] Huang C.L., Chang C.J., Chen K.C., Cheng S.W., Liu J.K., Lee T.M. (2022). Effect of thermocycling-induced stress on properties of orthodontic NiTi wires. J Dent Sci.

[16] Inokoshi M., Kameyama A., De Munck J., Minakuchi S., Van Meerbeek B. (2013). Durable bonding to mechanically and/or chemically pre-treated dental zirconia. J Dent.

[17] Camargo B., Willems E., Jacobs W. (2022). 3d printing and milling accuracy influence full-contour zirconia crown adaptation. Dent Mater.

[18] Dapieve K.S., Machry R.V., Cadore-Rodrigues A.C. (2022). Cyclic fatigue vs static loading for shear bond strength test of lithium disilicate and dentin substrates: a comparison of resin cement viscosities. Dent Mater.

[19] Ilday N., Seven N. (2011). The influence of different fiber-reinforced composites on shear bond strengths when bonded to enamel and dentin structures. J Dent Sci.

[20] Archangelo K., Guilardi L., Campanelli D., Valandro L., Borges A. (2019). Fatigue failure load and finite element analysis of multilayer ceramic restorations. Dent Mater.

[21] Dolev E., Bitterman Y., Meirowitz A. (2019). Comparison of marginal fit between CAD-CAM and hot-press lithium disilicate crowns. J Prosthet Dent.

[22] Gönülol N., Ertaş E., Yılmaz A., Çankaya S. (2015). Effect of thermal aging on microleakage of current flowable composite resins. J Dent Sci.

[23] McLean J.W., Fraunhofer J.A. (1971). The estimation of cement film thickness by an in vivo technique. Br Dent J.

[24] Elsaka S.E. (2015). Repair bond strength of resin composite to a novel CAD/CAM hybrid ceramic using different repair systems. Dent Mater J.

[25] Trindade F.Z., Kleverlaan C.J., Silva LHd (2016). Effect of mechanical cycling and ceramic type on restoration-dentin bond strength. Operat Dent.

[26] Isolan C.P., Valente L.L., Münchow E.A. (2014). Bond strength of a universal bonding agent and other contemporary dental adhesives applied on enamel, dentin, composite, and porcelain. Appl Adhes Sci.

[27] Fornazari I.A., Wille I., Meda E.M., Brum R.T., Souza E.M. (2017). Effect of surface treatment, silane, and universal adhesive on microshear bond strength of nanofilled composite repairs. Oper Dent.

[28] Kanzow P., Piecha L., Biermann J., Wiegand A. (2020). Repair surface conditioning measures affect enamel and dentin bond strength. Oper Dent.

[29] Franz A., Lettner S., Watts D.C., Schedle A. (2022). Should statistical analysis of bond-strength data include or exclude cohesive failures?. Dent Mater.

[30] Kim S.H., Choi Y.S., Kang K.H., Att W. (2022). Effects of thermal and mechanical cycling on the mechanical strength and surface properties of dental CAD-CAM restorative materials. J Prosthet Dent.

[31] Kiomarsi N., Saburian P., Chiniforush N., Karazifard M.J., Hashemikamangar S.S. (2017). Effect of thermocycling and surface treatment on repair bond strength of composite. J Clin Exp Dent.

[32] De Abreu R.A.M., Pereira M.D., Furtado F., Prado G.P.R., Mestriner W., Ferreira L.M. (2014). Masticatory efficiency and bite force in individuals with normal occlusion. Arch Oral Biol.

